# *De novo* transcriptome analysis of high-salinity stress-induced antioxidant activity and plant phytohormone alterations in *Sesuvium portulacastrum*

**DOI:** 10.3389/fpls.2022.995855

**Published:** 2022-09-23

**Authors:** YiQing Chen, Yan Zhou, Yuyi Cai, Yongpei Feng, Cairong Zhong, ZanShan Fang, Ying Zhang

**Affiliations:** ^1^Hainan Academy of Forestry, Hainan Mangrove Research Institute, Haikou, China; ^2^Mangrove Institute, Lingnan Normal University, Zhanjiang, China

**Keywords:** *Sesuvium portulacastrum*, plant phytohormones, antioxidant activity, transcription factor, salt stress

## Abstract

*Sesuvium portulacastrum* has a strong salt tolerance and can grow in saline and alkaline coastal and inland habitats. This study investigated the physiological and molecular responses of *S. portulacastrum* to high salinity by analyzing the changes in plant phytohormones and antioxidant activity, including their differentially expressed genes (DEGs) under similar high-salinity conditions. High salinity significantly affected proline (Pro) and hydrogen peroxide (H_2_O_2_) in *S. portulacastrum* seedlings, increasing Pro and H_2_O_2_ contents by 290.56 and 83.36%, respectively, compared to the control. Antioxidant activities, including superoxide dismutase (SOD), peroxidase (POD), and catalase (CAT), significantly increased by 83.05, 205.14, and 751.87%, respectively, under high salinity. Meanwhile, abscisic acid (ABA) and gibberellic acid (GA_3_) contents showed the reverse trend of high salt treatment. *De novo* transcriptome analysis showed that 36,676 unigenes were matched, and 3,622 salt stress-induced DEGs were identified as being associated with the metabolic and biological regulation processes of antioxidant activity and plant phytohormones. POD and SOD were upregulated under high-salinity conditions. In addition, the transcription levels of genes involved in auxin (*SAURs* and *GH3*), ethylene (*ERF1*, *ERF3*, *ERF114*, and *ABR1*), ABA (*PP2C*), and GA_3_ (*PIF3*) transport or signaling were altered. This study identified key metabolic and biological processes and putative genes involved in the high salt tolerance of *S. portulacastrum* and it is of great significance for identifying new salt-tolerant genes to promote ecological restoration of the coastal strand.

## Introduction

Salinity often has a significant effect on the survival and growth of plants. Salt stress causes ionic and osmotic stresses, which inhibits photosynthesis, protein synthesis, osmoregulation, and energy and lipid metabolism in plants (Zhu, 2001; Liska et al., 2004). Plants regulate many biological pathways to adapt to salt stress, including antioxidant defense systems and the perception and transduction of plant phytohormone signals (Liska et al., 2004; Hasanuzzaman et al., 2021). The antioxidant defense system (enzymatic and non-enzymatic systems) protects the plant from salt-induced oxidative damage by detoxifying the reactive oxygen species (ROS) and also by maintaining the balance of ROS generation under salt stress (Hasanuzzaman et al., 2021). The enzymatic system including superoxide dismutase (SOD), catalase (CAT), and peroxidase (POD) can scavenge ROS to protect the plant from oxidative stress damage, which is proved to act in reducing ROS under salt stress (Hasanuzzaman et al., 2020). Plant phytohormones including abscisic acid (ABA), auxins, cytokinins, gibberellic acid (GA), and ethylene are chemicals in small amounts but have a great impact to help plants to adapt under salinity (Ribba et al., 2020). Phytohormone signaling pathways also interact with ROS scavenging systems to mount a stress response (Hasanuzzaman et al., 2018). Several physiological processes are regulated by auxins during plant development under salt stress (Ribba et al., 2020). Auxin positively regulates antioxidant enzymes (SOD, CAT, and POD) and confers effective ROS detoxification, thus improving plant abiotic stress resistance (Shi et al., 2014). ABA-dependent pathways may be positively regulated by auxins in response to abiotic stress (Liu et al., 2013; Shi et al., 2014). The ABA-induced hydrogen peroxide (H_2_O_2_; non-radical ROS) generation activates mitogen-activated protein kinase (MAPK), which in turn induces the expression level and activity of antioxidant enzymes to scavenge ROS (Zhang A. et al., 2006; Lu et al., 2009; Fahad et al., 2015). A previous study has suggested that GA plays a significant role in regulating the stress response process (Kirungu et al., 2019). Growth inhibition caused by salt stress may be an active adaptation mechanism in plants that regulates GA levels to achieve an optimal growth rate in response to environmental changes. The specific concentration of GA_3_ can regulate the metabolic process as a function of sugar signaling and antioxidative enzymes under salt stress (Mohammed, 2007; Iqbal et al., 2011). ABA and GA can affect the germination rate of saline plants, and there is a significant change in GA concentration in plants under abiotic stress, indicating an interaction between ABA and GA (Yu et al., 2020).

*Sesuvium portulacastrum* (Aizoaceae, *Sesuvium*), a perennial herb with thick, smooth stems (Alsherif et al., 2021), is a well-known halophyte plant with strong drought and salt tolerance that can grow in saline and alkaline coastal and inland habitats (El-Awady et al., 2015). *Sesuvium* is a “salt accumulator” plant that accumulates high concentrations of salts in its cells and tissues and overcomes salt toxicity by developing succulence (Meng et al., 2018). *S. portulacastrum* exhibits high photosynthetic activity, succulence, biomass, and antioxidant defense mechanisms at the cellular, organ, and plant levels (Lokhande et al., 2013; Muchate et al., 2016). In China, it is found in mangrove forests. To study their salt response mechanism, seedlings were cultivated in high salt concentrations of 500 mM for 28 days (Nikalje et al., 2018), 500 mM and 600 mM for 28 days (Kannan et al., 2013), and 400 and 600 mM for 14 days (Peng et al., 2019). These studies have focused on propagation, photosynthesis, ion homeostasis, antioxidant defense, accumulation mechanisms, salt-responsive genes, and proteins of *S. portulacastrum* under salt stress (Rabhi et al., 2010; Lokhande et al., 2013; Yi et al., 2014; Nikalje et al., 2018; Fan et al., 2019; Peng et al., 2019; He et al., 2021), but the signaling pathway involved in salt stress is not known at the transcriptome level, even though one transcriptome profiling study was reported for the same genus plant *Sesuvium verrucosum* under salinity stress (Thayale Purayil et al., 2022).

The understanding of plant responses to salt stress has expanded with the development of next-generation sequencing technologies. Studies on plant genomics have extensively used transcriptome sequencing techniques (Das et al., 2020; Meera and Augustine, 2020; Elnaggar et al., 2021). Therefore, a high-salinity response mechanism was studied using the RNA-seq technology to screen for salt-tolerance genes. Differentially expressed genes (DEGs) are predominantly enriched in cellular amino acid catabolic processes and responses to ROS biosynthetic processes (Yue et al., 2021). Plant hormone signal transduction genes have been discovered in salt-treated *Solenostemma argel* (Ahmad et al., 2021).

Transcription factor genes such as MYBs, NAC, B3, and AP2/ERF were found to be differentially expressed and helped the plant adapt to high-salinity conditions in Arabidopsis (Ma et al., 2022) and other non-model plants, *Kandelia candel*, *Bruguiera gymnorhiza* (Basyuni et al., 2012), and *Sonneratia caseolaris* (Zhou et al., 2022). In this study, we analyzed the physiological response of *S. portulacastrum* to salt stress. Meanwhile, *de novo* transcriptome patterns of *S. portulacastrum* in the leaves identified genes associated with high-salinity tolerance. The results of this study elucidate the mechanisms underlying high-salinity tolerance in *S. portulacastrum* and provide an important role in coastal soil remediation.

## Materials and methods

### Plant materials and different NaCl treatments

*Sesuvium portulacastrum* plants were collected from mangrove areas (110.39° N, 21.27° E) in Zhanjiang, Guangdong Province, South China, and propagated by cutting. Notably, 6-cm-long stem segments with two nodes and two opposite leaves on the top node were taken from mother plants and cultivated in tap water under natural conditions for 30 days. Then, the plants were cultivated weekly with a half-strength modified Hoagland nutrient solution (Wang et al., 2012; Yi et al., 2014) and with different NaCl concentrations of 0 (control), 100, 200, 400, 600, and 800 mmol/L. A month later, leaves from each treatment were collected for further experiments. The sample treated with 800 mmol/L NaCl perished. Each sample contained three biological replicates.

### Determination of proline, hydrogen peroxide, antioxidant enzyme activity, and plant phytohormone content

Proline (Pro) content was determined following a previous study using the ninhydrin colorimetric method, with some modifications (Rosen, 1957). H_2_O_2_ content was determined spectrophotometrically at 410 nm, as described previously (Zhou et al., 2017; Ali et al., 2019). To assay SOD (EC 1.15.1.1), the inhibition of the photochemical reduction of nitro-blue tetrazolium was monitored, as described previously (El-Shabrawi et al., 2010). POD (EC1.11.1.7.) activity was measured at 470 nm, as described by Zhou et al. (2017). CAT (EC 1.11.1.6.) activity was assayed at 240 nm using the method described by Hasanuzzaman et al. (2011). Gas chromatography-mass spectrometry (GC-MS) analysis was used to determine the contents of auxin (IAA) and ABA in a previous study (Zörb et al., 2013). The samples were freeze-dried and ground to a powder to determine the gibberellin (GA_3_) content by GC-MS following the method of a previous study (Ptošková et al., 2022).

### RNA extraction, library preparation, and Illumina NovaSeq 6000 sequencing

Total RNA was extracted from the leaf tissues using TRIzol^®^ Life Reagent (Invitrogen, Carlsbad, CA, USA). RNA was quantified, and a sequencing library was built using the present method (Zhang et al., 2020). RNA purification, reverse transcription, library construction, and sequencing were performed following the manufacturer’s instructions (Illumina, San Diego, CA, USA and sequencing). The Illumina TruSeq™ RNA sample preparation kit (San Diego, CA, USA) was used to prepare the RNA-seq libraries. Briefly, oligo-dT-attached magnetic beads were used to purify poly(A) mRNA and further synthesize double-stranded cDNA. Later, end-repair was performed on the synthesized cDNA, and the addition of “A” bases was followed by PCR amplification. A total of 6 RNA-seq libraries were sequenced using an Illumina NovaSeq 6000 sequencer (Illumina, San Diego, CA, USA). Accession number PRJNA859253 was assigned to the raw reads generated for this study in the NCBI database.

### *De novo* assembly and annotation

*De novo* assembly was performed on cleaned data using Trinity (Grabherr et al., 2011). BLASTX with an *e*-value of lower than 1.0 × 10^–5^ was used to predict the unigenes from the protein non-redundant (NR), Clusters of Orthologous Groups (COG), Kyoto Encyclopedia of Genes and Genomes (KEGG), and NCBI databases. The BLAST2GO program was used to obtain gene ontology (GO) annotations of uniquely assembled transcripts to describe potentially associated biological processes, molecular functions, and cellular components (Conesa et al., 2005). KEGG was used for the metabolic pathway analysis (Ogata et al., 1999).

### Differentially expressed genes identification and functional enrichment

Differentially expressed genes of two different samples were identified by calculating the expression levels of each transcript using the transcripts per million reads method. Gene abundance was calculated using RSEM (Li and Dewey, 2011). EdgeR (Robinson et al., 2010) was used to analyze gene expression and DEGs with a | log2FC| > 1 and *Q*-value ≤ 0.05. GO and KEGG analyses were enriched in DEGs to identify relevant pathways and functions (Xie et al., 2011). A Bonferroni-corrected *P*-value of ≤ 0.05 was used when compared to the background comprising the whole transcriptome.

### Quantitative real-time PCR analysis

Quantitative real-time PCR analysis uses our present method (Zhang et al., 2020). The program Primer Premier version 5.0 was used to create the primers (Supplementary Table 1). The length of the amplified PCR products ranged from 80 to 400 bp. The relative expression levels of the genes in various samples were estimated using the 2^–Δ^ ^Δ^ ^Ct^ method, with actin as a reference to calculate the relative expression of the DEGs.

### Statistical analysis

Data from each group were analyzed individually using the SPSS software (version 19.0). Tukey’s test value of *P* < 0.05 indicated a statistical significance, and significant differences were indicated by different letters above bars.

## Results

### Effects of salt stress on Pro, H_2_O_2_ content, and antioxidant enzyme activities

**FIGURE 1:**
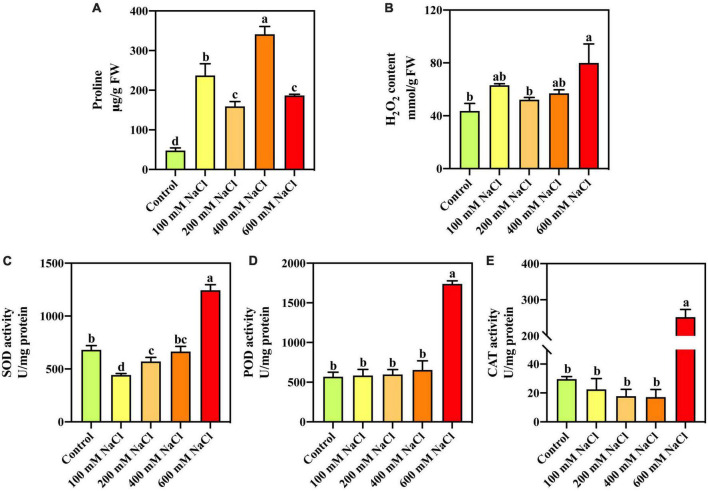
The proline **(A)**, H_2_O_2_ content **(B)**, and activities of SOD **(C)**, POD **(D)**, and CAT **(E)** in the leaves of *S. portulacastrum* under salt stress.

Compared with the control, Pro content was significantly increased under different NaCl concentrations (Figure 1A). With increasing salt concentration, the Pro content first decreased, then increased, and finally decreased. Pro content increased by 613.25% under 400 mM NaCl treatment and by 290.56% under the highest concentration (600 mM) NaCl treatment. The H_2_O_2_ content was significantly increased by 83.36% under the 600 mM NaCl treatment compared with the control (Figure 1B). Compared with the control, SOD, POD, and CAT activities were significantly increased by 83.05, 205.14, and 751.87%, respectively, under the 600 mM NaCl treatment (Figures 1C–E). The low-concentration NaCl treatment did not significantly affect POD and CAT activities in *S. portulacastrum*.

### Effects of salt stress on IAA, ABA, and GA_3_ content

**FIGURE 2:**
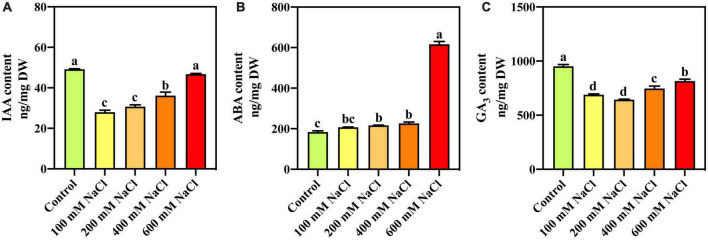
The content of IAA **(A)**, ABA **(B)**, and GA_3_ **(C)** in the leaves of *S. portulacastrum* under salt stress.

With an increase in salt concentration, IAA content decreases with high salinity but piques to a normal level as part of homeostasis (Figure 2A). Compared with the control, the ABA content was significantly increased under different NaCl treatments (Figure 2B). The ABA content in *S. portulacastrum* leaves treated with 600 mM NaCl was 3.36 times that of the control. Compared with the control, GA_3_ content was significantly decreased under the different NaCl treatments (Figure 2C). The GA_3_ content in *S. portulacastrum* leaves treated with 600 mM NaCl was used as a control.

### RNA sequencing, *de novo* assembly, and transcriptome annotation

Compared with the control, *S. portulacastrum* seedling leaves under the 600 mM NaCl treatment had higher SOD, POD, and CAT activities to scavenge H_2_O_2_ and higher IAA and ABA contents in response to high salinity. Thus, we performed a transcriptomic analysis of the leaves of *S. portulacastrum* seedlings following control and 600 mM NaCl treatments. A total of 38.19 Gb of data were obtained from six cDNA libraries. After trimming low-quality reads and adapters, 256,867,510 clean reads with a Q30 higher than 92.50% were obtained (Supplementary Table 2). In total, 217,969 transcripts and 123,007 unigenes were obtained, with an N50 length of 1,184 bp and an average length of 734.24 bp (Table 1). Unigene lengths of 200–500, 501–1,000 bp, 1,001–1,500, and > 1,500 bp accounted for 60, 21, 7, and 12%, respectively (Supplementary Table 3), and 23,431 unigenes had lengths of > 1,000 bp.

**TABLE 1:** Evaluation of the assembly result.

Type	Unigene	Transcript
Total number	123007	217969
Total base	90316943	194029261
Largest length (bp)	16885	16885
Smallest length (bp)	201	201
Average length (bp)	734.24	890.17
N50 length (bp)	1184	1452
E90N50 length (bp)	2301	1984
Fragment mapped percent (%)	59.45	76.63
GC percent (%)	39.89	40.46
TransRate score	0.28298	0.3722
BUSCO score	C:75.9% [S:74.4%; D:1.5%]	C:89.3% [S:27.5%; D:61.8%]

A total of 36,676 (30.80%) unigenes were matched to known the genes at least once in the listed databases. Of note, 25,478 (21.40%) and 30,893 (25.95%) unigenes were the best hits in the NR and COG databases, respectively, followed by 21,077 (17.70%) in the GO database, 25,715 (21.60%) in the Swiss-Prot database, 25,086 (21.07%) in the Pfam database, and 16,672 (14.00%) in the KEGG database (Table 2).

**TABLE 2:** Statistics of annotation results.

Type	Exp_Unigene number (percent)	Exp_Transcript number (percent)	All_Unigene number (percent)	All_Transcript number (percent)
GO	21077 (0.177)	59074 (0.2783)	21274 (0.1729)	59792 (0.2743)
KEGG	16672 (0.14)	43641 (0.2056)	16919 (0.1375)	44275 (0.2031)
COG	30893 (0.2595)	81785 (0.3853)	31253 (0.2541)	82796 (0.3799)
NR	25478 (0.214)	71730 (0.3379)	25704 (0.209)	72568 (0.3329)
Swiss-Prot	25715 (0.216)	68713 (0.3237)	25987 (0.2113)	69571 (0.3192)
Pfam	25086 (0.2107)	67173 (0.3165)	25351 (0.2061)	67980 (0.3119)
Total_anno	36676 (0.308)	93291 (0.4395)	37151 (0.302)	94498 (0.4335)
Total	119070 (1)	212259 (1)	123007 (1)	217969 (1)

**FIGURE 3:**
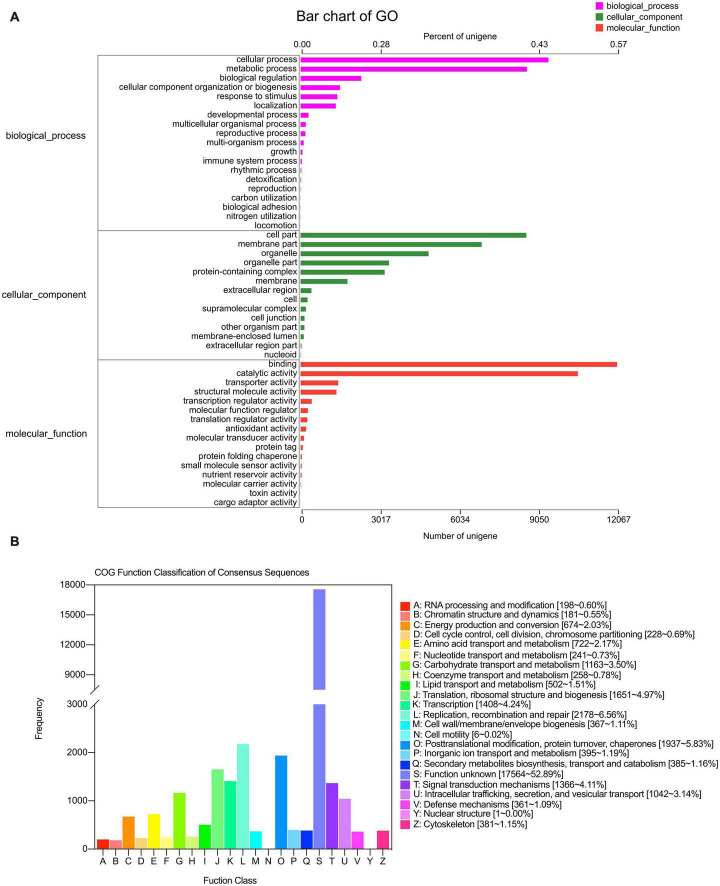
GO **(A)** and COG **(B)** annotation of transcriptome in the leaves of *S. portulacastrum* under high salinity.

A total of 21,077 unigenes were the best hits in the GO database and were enriched for 49 GO terms that classified as biological processes, cellular components, and molecular functions. In biological processes, most of the unigenes were enriched for “cellular process” (9,443), “metabolic process” (8,624), “biological regulation” (2,312), “cellular component organization or biogenesis” (1,497), “response to stimulus” (1,400), and “localization” (1,340) terms, accounting for 36.93, 33.72, 9.04, 5.85, 5.47, and 5.23%, respectively. The other two processes accounted for less than 9% of the gene enrichment. For the cellular component category, the largest subcategories were “cell part” (8,605; 28.63%), “membrane part” (6,900; 22.96%), “organelle” (4,877; 16.23%), “protein-containing complex” (3,200; 10.65%), and “membrane” (1,784; 5.94%). Regarding molecular function, most of the unigenes were enriched for “binding” (12,067; 44.82%) and “catalytic activity” (10,568; 39.25%) (Figure 3A).

### Analysis of differentially expressed genes (differentially expressed genes) and gene co-expression clusters

**FIGURE 4:**
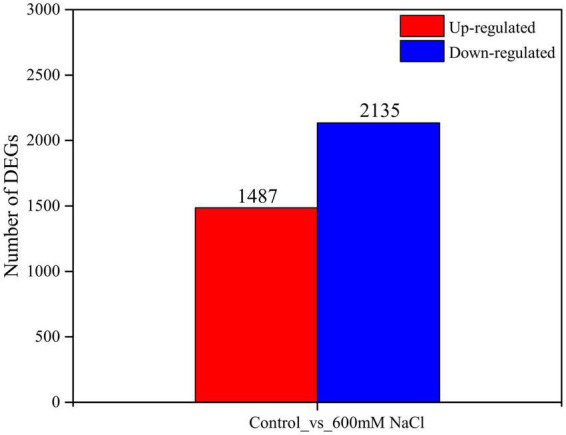
The number of DEGs in the leaves of *S. portulacastrum* under high salinity.

**FIGURE 5:**
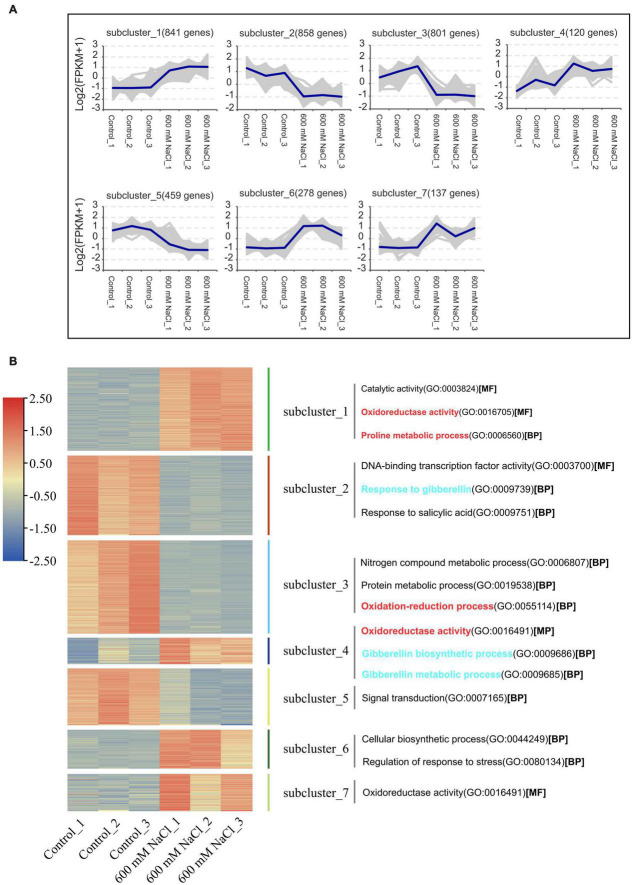
Gene co-expression clusters **(A)** and heatmap analysis **(B)** of DEGs in the leaves of *S. portulacastrum* under high salinity.

A *de novo* assembled reference transcriptome of *S. portulacastrum* was aligned with the RNA-seq reads from the 600 mM NaCl treatment group and the control group; the average mapping rate was 78.44% (Supplementary Table 4). The Pearson’s correlation coefficient between the three biological replicates of the 600 mM NaCl treatment and the control ranged from 0.925 to 1.0 (Supplementary Figure 1). Principal component analysis indicated that the control and 600 mM NaCl treatments displayed distinct transcriptome characteristics (Supplementary Figure 2). A total of 10 clusters of gene co-expression patterns were identified, including a total of 3,622 DEGs in the control vs. 600 mM NaCl comparison (Figure 4 and Supplementary Datasets 1–3). The genes in Clusters 1, 4, 6, and 7 were significantly upregulated in the 600 mM NaCl group compared to the control group, with 841, 120, 278, and 137 genes, respectively (Figure 5A and Supplementary Dataset 1). The GO analysis showed that these upregulated genes were involved in catalytic activity, oxidoreductase activity, Pro metabolic process, gibberellin biosynthetic and metabolic processes, cellular biosynthetic processes, and regulation of stress response. The genes were significantly downregulated in Clusters 2 (858), 3 (801), and 5 (459) under the 600 mM NaCl treatment compared to the control (Supplementary Dataset 2). These genes were associated with DNA-binding transcription factor activity and response to gibberellin and salicylic acid and were involved in nitrogen compound and protein metabolic processes, oxidation-reduction processes, and signal transduction (Figure 5B).

**FIGURE 6:**
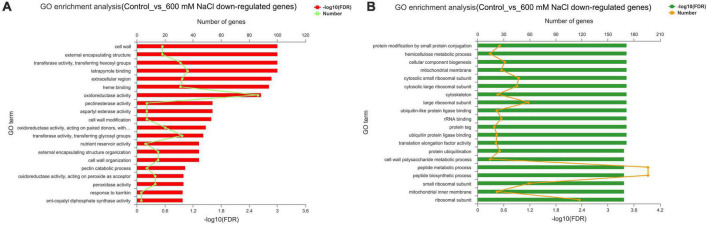
GO enrichment analysis of DEGs in the leaves of *S. portulacastrum* under high salinity. **(A)** Upregulated genes, **(B)** downregulated genes.

The upregulated genes (1,487) in the control vs. 600 mM NaCl were significantly enriched in oxidoreductase activity (GO:0016491), carbohydrate metabolic process (GO:0005975), structural molecule activity (GO:0005198), catabolic process (GO:0009056), and tetrapyrrole binding (GO:0046906). Notably, many pathways were related to response to stress, such as POD activity (GO:0004601), antioxidant activity (GO:0016209), ROS metabolic process (GO:0072593), and Pro metabolic process (GO:0006560). Many pathways were related to plant phytohormones, response to auxin (GO:0009733), and gibberellin biosynthetic and metabolic processes (GO:0009686; GO:0009685) (Figure 6A and Supplementary Dataset 4). The downregulated genes (2135) were significantly enriched in the organic substance metabolic process (GO:0071704), cellular metabolic process (GO:0044237), primary metabolic process (GO:0044238), macromolecule metabolic process (GO:0043170), and organelles (GO:0043226) (Figure 6B and Supplementary Dataset 5).

### Responses of transcription factors, plant phytohormones, and antioxidant activity to salt stress

**FIGURE 7:**
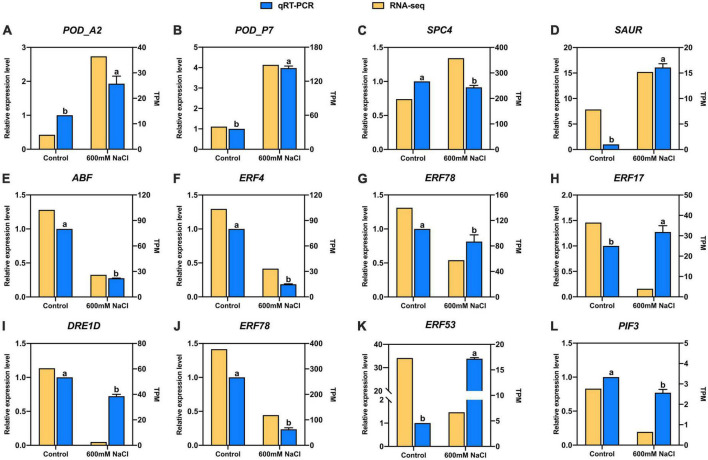
Expression pattern validation of 12 selected DEGs in *S. portulacastrum* determined by RNA-seq and qRT-PCR. **(A)** POD_A2, **(B)** POD_P7, **(C)** SPC4, **(D)** SAUR, **(E)** ABF, **(F)** ERF4, **(G)** ERF78, **(H)** ERF17, **(I)** DRE1D, **(J)** ERF78, **(K)** ERF53, and **(L)** PIF3.Values are means ± SD (*n* = 3). Values with a different letter within a sampling date are significantly different (*P* < 0.05).

The candidate 12 DEGs were randomly selected to verify the accuracy of the RNA-seq data of *S. portulacastrum* under high-salinity conditions. Furthermore, qRT-PCR primers were used to determine expression levels (Supplementary Table 1). These results indicated that the expression patterns of these genes were highly consistent with the results of RNA-seq analysis (Figure 7). The results indicated that the RNA-seq data had a high level of reliability and that these DEGs were suitable for further analysis in *S. portulacastrum* under high salinity.

**FIGURE 8:**
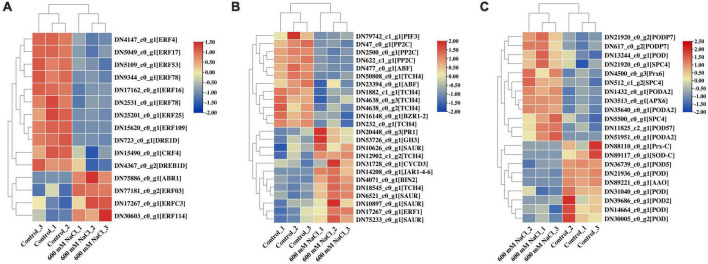
Heatmap analysis of AP2 gene family **(A)**, plant phytohormones **(B)**, and antioxidant activity **(C)** related to DEGs in the leaves of *S. portulacastrum* under high salinity.

In the assembled transcriptome of *S. portulacastrum*, 802 TFs were predicted. Among them, more than 40 members belonged to the MYB, AP2/ERF, bHLH, C2C2, NAC, and B3 families (Supplementary Figure 3). In addition, we identified a number of phytohormone transport or synthesis-related genes in the DEGs of *S. portulacastrum* after high salinity, such as four AP2/ERF gene families that were upregulated, and 11 AP2/ERF gene families that were downregulated (Figure 8A and Supplementary Dataset 6). Auxin-responsive protein SAURs and GH3 auxin-responsive promoters were also upregulated (Figure 8B). One gene related to the ethylene response factor (ERF1) was upregulated. Three protein phosphatases 2C (PP2C) and one ABA-insensitive were downregulated, which were involved in ABA signal transduction. One transcription factor, PIF3, was downregulated, which is associated with gibberellin metabolism (Figure 8B). DEGs related to antioxidant activity were identified in *S. portulacastrum* under 600 mM NaCl treatment, such as PODs, L-ascorbate peroxidase (APX), and peroxiredoxin (Prx) (Figure 8C). A total of 10 PODs, one Prx, and one APX gene were upregulated; and six POD genes, one Prx, and one SOD were downregulated (Figure 8C).

## Discussion

It is becoming increasingly common for soils to become salinized worldwide, negatively influencing plant growth (Parmar et al., 2020). The use of salt-tolerant plants reduces seawater erosion on the coast by creating an environment that facilitates plant growth, aids in the development of a bioreactor system, and assists in the bioremediation process (Duarte et al., 2013; Fariña et al., 2018). The herb *S. portulacastrum* can tolerate high salt levels without salt glands and bladders in its succulent leaves, indicating the existence of a specialized salt tolerance mechanism (Yi et al., 2014). Although salt-responsive physiological effects have been documented previously in this plant, insight into salinity-induced antioxidant activity and phytohormone alterations is essential to further understand the salt adaptation mechanisms (Lokhande et al., 2013; Muchate et al., 2016; Nikalje et al., 2018). To date, the current understanding of the complex molecular mechanisms underlying salt tolerance in *S. portulacastrum* remains limited (Fan et al., 2019). High-throughput RNA sequencing is required to identify candidate genes involved in salt stress tolerance and will also facilitate a better understanding of the salt stress tolerance mechanisms in *S. portulacastrum* (Bhadauria, 2017). In addition, it has been used in the Chinese traditional medicine for centuries. Despite this, less research has been conducted on the molecular basis of physiological functions (Rabhi et al., 2010; He et al., 2021; Zhang C. et al., 2022). Salt stress resistance has not been studied at the transcriptome level. Therefore, investigating the molecular mechanisms of acclimation to salt stress and identifying new salt-tolerant genes that promote ecological restoration of the coastal strand are essential.

### *Sesuvium portulacastrum* could rapidly scavenge ROS to salinity stress at physiological and transcriptome levels

Osmotic stress and ion toxicity caused by salt stress can cause a series of secondary stresses, such as oxidative stress, significantly increasing ROS levels in plants. Various adaptations have been observed in *S. portulacastrum* in response to salt stress, including changes in morphological and anatomical growth, water use efficiency, and physiological and biochemical changes (Moseki and Buru, 2010; Lokhande et al., 2013). *S. portulacastrum* has a steady adaptation to unfavorable conditions because of its high antioxidant capacity, which also enhances its stress tolerance (Rabhi et al., 2010). Pro is the main osmotic regulator in many non-salt plants and has an essential function in maintaining the water content and enzyme activity of cells (Ullah et al., 2018; Jannesar et al., 2020). The intracellular Pro content of *S. portulacastrum* significantly increased under high salt stress, indicating that *S. portulacastrum* also adopts the same osmotic adjustment mechanism to adapt to external salt stress. The increased H_2_O_2_ illustrated that excess ROS was produced in plant cells under salt stress (Sachdev et al., 2021). Plants eliminate excess ROS produced by salt-induced stress by initiating an enzymatic system, which mainly results in an increase in antioxidant enzyme activity (Shafi et al., 2015). SOD, POD, and CAT activities were significantly increased under high-salinity conditions in *S. portulacastrum*.

Many genes involved in oxidoreductase activity and antioxidant activity were significantly enriched in DEGs in the transcriptome of *S. portulacastrum* exposed to high salinity. GO enrichment analysis revealed that these genes were related to antioxidant activity. In salt-stressed plants, SOD, POD, APX, and Prx play critical roles in maintaining redox homeostasis. One manifestation of the disruption of cellular redox homeostasis is the accumulation of oxidized Prxs (Poynton and Hampton, 2014). This study found that *PODs* (*PODP7*, *PODA2*, *POD*, and *POD57*) and *Prx6* were significantly upregulated, and *SOD-C* was significantly downregulated. POD family members respond differently to salt stress. The expression of seven POD genes increased after salt stress, whereas the transcripts of two POD genes decreased in grapevines (Xiao et al., 2020). The high expression levels of *PODs* contributed to the conference on salt tolerance in *S. portulacastrum*, indicating the same trend as the physiological level.

### Phytohormone in *Sesuvium portulacastrum* responses to high salinity at physiological and transcriptome levels

Salt stress affects plants at all stages of their life cycle, from inhibiting seed germination to modifying plant growth and development. Water deficits, ion toxicity, and ion imbalance are the three main reasons that salt negatively affects plants (Ribba et al., 2020). Thus, plants have evolved mechanisms to cope with stress, including stress-sensing and downstream response mechanisms controlled primarily by plant hormones. High-salinity exposure has been associated with the release of ABA, auxins, GA, and ethylene, playing essential roles (Egamberdieva et al., 2018). Several studies have demonstrated that plants under salt stress have lower auxin levels and decreased expression of auxin transporters (Liu et al., 2015). In this study, IAA content was controlled under high-salinity conditions. According to earlier research, genes involved in auxin transport and responsiveness, such as SAURs, were increased in response to severe salt stress (Wu et al., 2012). *Gh_A08G1120* (*GH3.5*) is upregulated in cotton under salt stress (Kirungu et al., 2019). Plant responses to salt stress are strongly influenced by ethylene, the most significant stress-responsive hormone (Tao et al., 2015). The expression levels of genes in *S. portulacastrum*, which are involved in the ethylene response, as well as ethylene signaling, changed significantly. Genes related to ethylene response and transcription factors, such as *ERFs*, were downregulated in response to salt stress. *ERF1*, *ERF3*, *ERF114*, and *ABR1* were upregulated under high-salinity conditions in *S. portulacastrum*. Overexpression of *ERF1* improves salinity tolerance by amplifying the ROS-activated MAPK cascade signal (Schmidt et al., 2013). In response to salt and drought stress, *TaERF3* positively regulates wheat adaptation (Rong et al., 2014). Overexpression of *ERF114* enhances resistance to disease by positively modulating lignin and salicylic acid accumulation induced by PevD1 (Li et al., 2022). The ability of *ERF114* in *S. portulacastrum* to help plants under salt stress requires further research. The expression of *ABR1* was responsive to ABA and high salt stress. It has been shown that ABA induces hypersensitivity to seed germination and root growth in the absence of *ABR1* (Pandey et al., 2005). Promoter analysis of *the BjABR1* gene showed that there were many hormones and stress-related *cis*-acting elements, and expression analysis of this gene showed that it was induced under high salt stress (Xiang et al., 2018).

Abscisic acid is defined as a stress hormone due to its rapid accumulation in response to stress and its role in mediating many stress responses that assist plants in survival (Zhang J. et al., 2006). ABA was high in response to high salinity in *S. portulacastrum*. ABA has been shown to inhibit ethylene production during stress as part of its function (Zhang J. et al., 2006). PP2C genes have been shown to regulate ABA signaling *via* modulation of SnRK or MAPK kinase activity during abiotic stress responses (Yang et al., 2018). *PP2C* genes were downregulated under high-salinity conditions in *S. portulacastrum* at the transcriptome level. *PP2C1* is a member of the PP2C family. *BpPP2C1* loss of function resulted in lower salt tolerance, but its overexpression increased SOD and POD activities to improve salt tolerance (Xing et al., 2021). *S. portulacastrum*, as a halophyte, has different *PP2C* gene expression levels compared to the control plants. *CsPP2C* genes in different groups have different responses to different stressors (Zhang G. et al., 2022), and these results provide a reference for the study of *PP2C* under salt stress. Salt stress has been alleviated using GA_3_ as a growth regulator, which has been utilized to accelerate the growth of wheat and rice (Forghani et al., 2018). GA_3_ decreased under different salt treatments but was high in *S. portulacastrum* treated with 600 mM NaCl. GO enrichment analysis revealed that the genes involved in gibberellin biosynthesis and metabolic processes were upregulated. In summary, *S. portulacastrum* can maintain its growth under salt stress, with GA playing a vital role (Zhu et al., 2021). In coordination with the circadian clock and GA_3_, phytochrome-interacting factor 3 (*PIF3*) modulates plant growth and development by activating the light-responsive transcriptional network genes. Overexpression of *ZmPIF3* in rice enhances its tolerance to salt stress without causing growth retardation (Gao et al., 2015). The interaction between plant phytohormones requires further study.

## Conclusion

Pro and H_2_O_2_ contents increased in *S. portulacastrum*. SOD, POD, and CAT activities also increased to decompose H_2_O_2_ to reduce ROS and minimize the oxidative stress caused by high salinity. Genes encoding antioxidant enzymes, such as *PODs* and *SOD*, were upregulated, and *Prx6* was downregulated in response to high salinity. ABA content increased and GA_3_ content decreased under high-salinity conditions in *S. portulacastrum*. At the transcriptome level, auxin signal receptors *SAURs* and *GH3* were upregulated in *S. portulacastrum* and played critical roles in its adaptation to high salinity. *ERF1*, *ERF3*, *ERF114*, and *ABR1*, associated with ethylene signaling, were upregulated in response to high salinity. *PP2C* and *PIF3*, which are involved in ABA and GA_3_ signal transduction, respectively, were downregulated under high-salinity conditions in *S. portulacastrum*. This study focused on the mechanism of salt tolerance at the physiological and transcriptome levels while laying the foundation for further study of the molecular mechanism of salt tolerance in *S. portulacastrum* and exploring new salt-tolerant genes.

## Data availability statement

The datasets presented in this study can be found in online repositories. The names of the repository/repositories and accession number(s) can be found below: https://www.ncbi.nlm.nih.gov/, PRJNA859253.

## Author contributions

YaZ defined the research theme and wrote the manuscript. YiZ and YiC designed methods and experiments, and carried out interpreted the results. YuC, YF, CZ, and ZF co-designed the experiments, carried out the laboratory experiments, and discussed the analyses and interpretation. All authors have read and approved the final manuscript.

## Supplementary material

Supplementary Figure 1 Heatmap and Pearson correlation coefficients for RNA-seq replicates of *S. portulacastrum* under high salinity. Three biological replicates are shown.

Supplementary Figure 2 The PCA of transcriptomes of *S. portulacastrum* under high salinity and control.

Supplementary Figure 3 The assembling transcriptome of *S. portulacastrum* revealed a number of different transcription factor families.
