# Supplementary material for: De novo transcriptome analysis of high-salinity stress-induced antioxidant activity and plant phytohormone alterations in Sesuvium portulacastrum
Source: Front Plant Sci. 2022 Sep 23;13:995855. doi: 10.3389/fpls.2022.995855 (PMC9540214; doi:10.3389/fpls.2022.995855)
Supplement: Supplementary file 1 [file Table_1.DOCX]

| Gene Name | Primer sequences（5'-3') | Length(bp) |
| --- | --- | --- |
| *Actin* | FP:TTCCGCAACATTCCCAGTAGGTATG  RP:CGCATAAGACACAATCCAACCACTG | 25  25 |
| *SAUR* | FP:TCAGCCCAAGAGTACGGATACGAG RP:AATCAGCACCACCACAACGAAGAG | 24  24 |
| *ABF* | FP:CACATCCTCTGTTTCACCAGTTCCC RP:CTTTATCATTCGCCTCTGCCGTCTC | 25  25 |
| *PP2C_1* | FP:TGGTCGAGAGGAAATGGAGGAGATG RP:AATCGCCACAATTAGCCACAACAAC | 25  25 |
| *GH3* | FP:GGACAGGAGGCAGCTACTCTACAG RP:GAGAACTGGGCGGGCTACTAGG | 24  22 |
| *PP2C_2* | FP:CCATCCCTCTCTCCTCCGATCAC RP:CCCGAGCAACAAAAGCGAAATAGTG | 23  25 |
| *PIF3* | FP:ATGATGATGCCGCCTGGAATGC RP:TCCCATAGCCCATGCCCATACC | 22  22 |
| *POD_A2* | FP:ACGAGATGCAGTGTCCTTGTTGG RP:TGCTTTGGCTTGCTGTTGTTGAATC | 23  25 |
| *POD_P7* | FP:TCTCTTCCCTCAGTTCTACGACCAC RP:TAGCCACAGCCTTAGCAACAACAG | 25  24 |
| *APX* | FP:CCCTCTCGTCTCTCACCTCTTTCTC RP:CTCCTTGATGTCTTCTCTGGCACTC | 25  25 |
| *SPC4* | FP:TGCTCTTGCTGCTCGTGATTCTG RP:AATGGTTGTTGCGTTGCTAAATGGG | 23  25 |
| *ERF4* | FP:CCGTGTCTGGCTTGGAACCTTC RP:GCCTTGGACCCACGAAACTCAC | 22  22 |
| *ERF78* | FP:CGTGGTCGAGGAGAGTGAGTGAG RP:GGGTGGAGCCAAGTTGAGATCAAG | 23  24 |
| *ERF17* | FP:ACAAGGGAGTGAGGAAGAGGAAGTG RP:GTCGTAGGAGCCAAGCCAGATTC | 25  23 |
| *DRE1D* | FP:GGCTGGGCGGAAGAAGTTCAAG RP:CTTATTGGGTTCACGGACCTCACAC | 22  25 |
| *ERF78* | FP:CACCAGCATCATCCGTACAGTCAG RP:TCAAATCAACCACCGAAGACGAGTC | 24  25 |
| *ERF53* | FP:GTTCAAGCTCCGAGGCGAGAATG RP:AGAATCCTCCTCTGGCTCTGGTTC | 23  24 |

Table S1.The primer sequences of DEGs by qRT-PCR analysis.
