# Supplementary material for: De novo transcriptome analysis of high-salinity stress-induced antioxidant activity and plant phytohormone alterations in Sesuvium portulacastrum
Source: Front Plant Sci. 2022 Sep 23;13:995855. doi: 10.3389/fpls.2022.995855 (PMC9540214; doi:10.3389/fpls.2022.995855)
Supplement: Supplementary file 2 [file Table_2.DOCX]

Table S2.Quality control of sequencing data.

| Sample | Raw reads | Raw bases | Clean reads | Clean bases | Error rate (%) | Q20(%) | Q30(%) | GC content (%) |
| --- | --- | --- | --- | --- | --- | --- | --- | --- |
| Control_1 | 46689842 | 7050166142 | 46147168 | 6849886499 | 0.0255 | 97.8 | 93.73 | 44.06 |
| Control_2 | 42559722 | 6426518022 | 42016404 | 6237377273 | 0.0256 | 97.74 | 93.61 | 44.31 |
| Control_3 | 43157526 | 6516786426 | 42649018 | 6340556822 | 0.0253 | 97.85 | 93.86 | 44.15 |
| 600mM NaCl_1 | 43241868 | 6529522068 | 42648390 | 6345221073 | 0.0267 | 97.36 | 92.50 | 44.59 |
| 600mM NaCl_2 | 42976628 | 6489470828 | 42349252 | 6316579925 | 0.0255 | 97.79 | 93.71 | 44.47 |
| 600mM NaCl_3 | 41642326 | 6287991226 | 41057278 | 6103103061 | 0.0254 | 97.81 | 93.81 | 45.12 |
