# Supplementary material for: De novo transcriptome analysis of high-salinity stress-induced antioxidant activity and plant phytohormone alterations in Sesuvium portulacastrum
Source: Front Plant Sci. 2022 Sep 23;13:995855. doi: 10.3389/fpls.2022.995855 (PMC9540214; doi:10.3389/fpls.2022.995855)
Supplement: Supplementary file 3 [file Table_3.DOCX]

Table S3.Length distribution of unigenes.

| Length | Number of Unigene | Percent of Unigene |
| --- | --- | --- |
| 200~500 | 73912 | 60% |
| 501~1000 | 25664 | 21% |
| 1001~1500 | 8631 | 7% |
| 1501~2000 | 5204 | 4% |
| 2001~2500 | 3563 | 3% |
| 2501~3000 | 2212 | 2% |
| 3001~3500 | 1440 | 1% |
| 3501~4000 | 853 | 1% |
| 4001~4500 | 553 | 0% |
| >4500 | 975 | 1% |
