# Supplementary material for: De novo transcriptome analysis of high-salinity stress-induced antioxidant activity and plant phytohormone alterations in Sesuvium portulacastrum
Source: Front Plant Sci. 2022 Sep 23;13:995855. doi: 10.3389/fpls.2022.995855 (PMC9540214; doi:10.3389/fpls.2022.995855)
Supplement: Supplementary file 4 [file Table_4.DOCX]

| Sample | Clean reads | Mapped reads | Mapped ratio |
| --- | --- | --- | --- |
| Control_1 | 23,073,584 | 18,089,782 | 78.40% |
| Control_2 | 21,008,202 | 16,341,233 | 77.79% |
| Control_3 | 21,324,509 | 16,412,234 | 76.96% |
| 600mM NaCl_1 | 21,324,195 | 16,847,646 | 79.01% |
| 600mM NaCl_2 | 21,174,626 | 16,760,769 | 79.15% |
| 600mM NaCl_3 | 20,528,639 | 16,283,093 | 79.32% |

Table S4.Comparison table of sequencing data and assembly results.
