# Supplementary figures and images for: De novo transcriptome analysis of high-salinity stress-induced antioxidant activity and plant phytohormone alterations in Sesuvium portulacastrum
Source: Front Plant Sci. 2022 Sep 23;13:995855. doi: 10.3389/fpls.2022.995855 (PMC9540214; doi:10.3389/fpls.2022.995855)

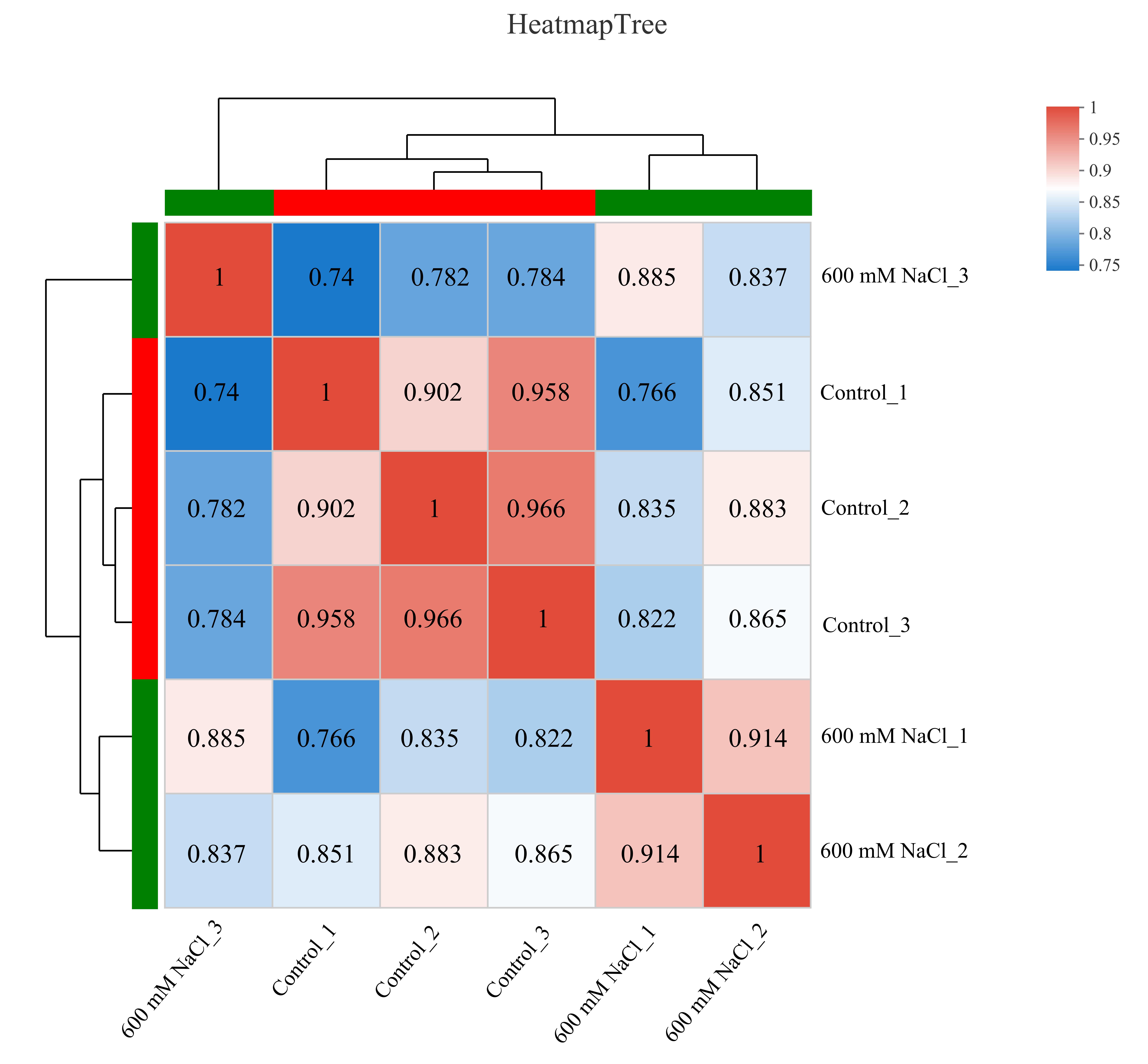

Supplement: Supplementary Figure 1 — Heatmap and Pearson correlation coefficients for RNA-seq replicates of S. portulacastrum under high salinity. Three biological replicates are shown. [file Image_1.JPEG]

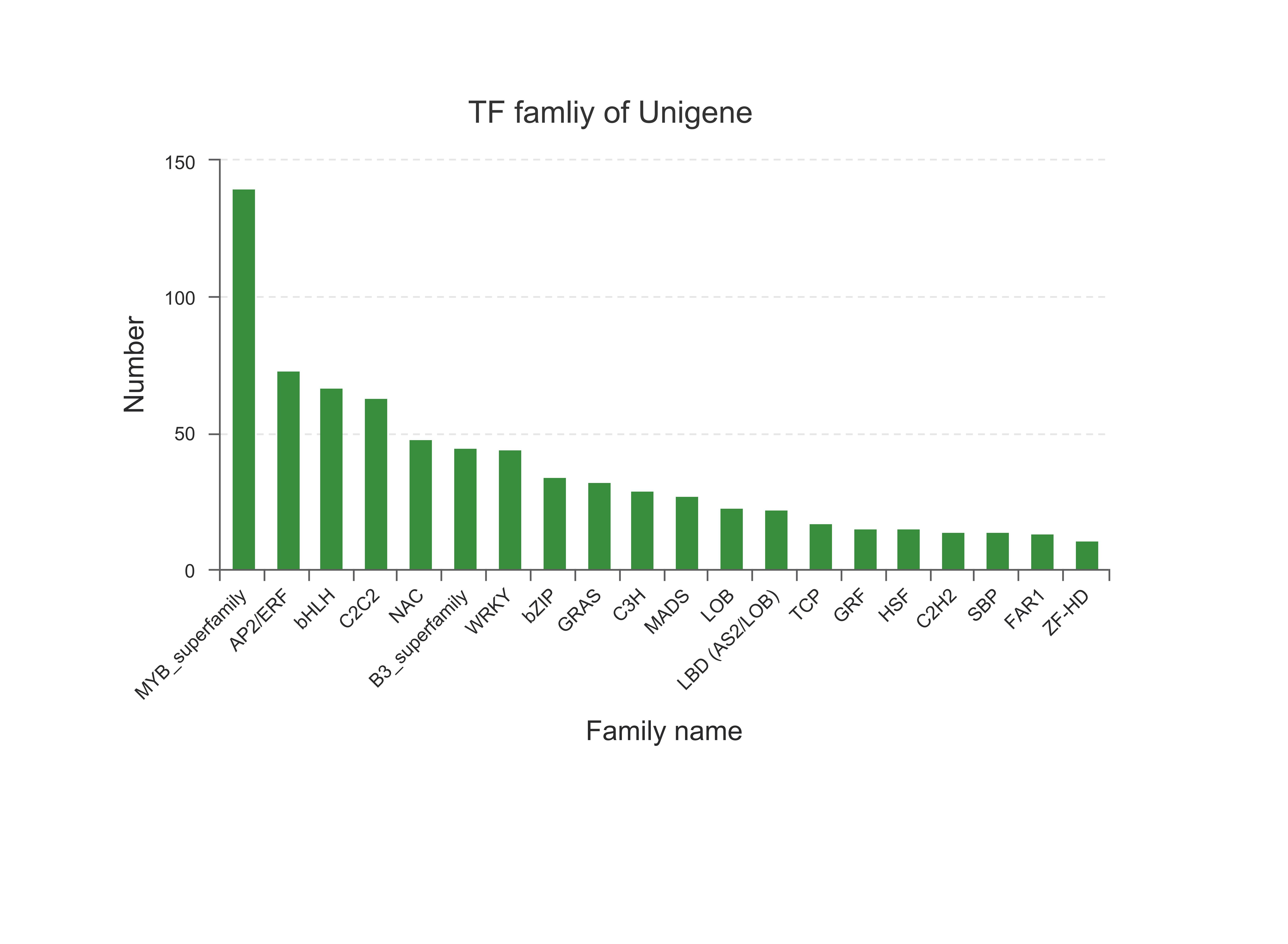

Supplement: Supplementary Figure 3 — The assembling transcriptome of S. portulacastrum revealed a number of different transcription factor families. [file Image_3.JPEG]
